# Artifactually elevated BUN values on the Boehringer Mannheim-Hitachi 737 and 705 automated chemistry analysers and the development of a kinetic BUN method

**DOI:** 10.1155/S1463924687000087

**Published:** 1987

**Authors:** William E. Neeley, Melissa Tyson, Kathleen O'Classen, Mary Gruber

**Affiliations:** 1 Division of Laboratory Medicine University of California School of Medicine University of California USA; 2 Veterans Administration Medical Center San Diego California 92161 USA


Journal of Automatic Chemistry/*Journal of Clinical Laboratory Automation*, Vol. 9, No. 1 (January–March 1987), pp. 41–43

### Artifactually elevated BUN values on the Boehringer Mannheim–Hitachi 737 and 705 automated chemistry analysers and the development of a kinetic BUN method

**William E. Neeley,[1,2] Melissa Tyson,[1] Kathleen O'Classen[2] and Mary Gruber[2]**

*[1] Division of Laboratory Medicine, University of California School of Medicine, University of California, USA; and [2] Veterans Administration Medical Center, San Diego, California 92161, USA*

### Introduction

The measurement of blood urea nitrogen (BUN) as serum or plasma urea is one of the most frequently ordered laboratory tests for evaluating renal functions. Conventional analysis of reactions of urea with diacetyl monoxime or urease with the Berthelot reaction [1] is rapidly giving way to the totally enzymatic reactions that are more convenient to perform and more easily adapted to modern instrumentation. However, the automated analysis of these reactions may yield falsely elevated BUN levels for some patients.

The authors' hospital recently acquired two new automated Hitachi analysers, Model 737 and 705 (Hitachi 737 Automatic Analyser and Hitachi 705 Automatic Analyzer [trademarks of Hitachi Ltd, Tokyo, Japan and marketed in the US by Boehringer Mannheim Diagnostics, Indianapolis, Indiana 46250]; aca, Automated Clinical Analyzer [trademark of Du Pont Instruments, Wilmington, Delaware 19898, USA]; SMAC, Sequential Multipole Analyzer with Computer [trademark of Technicon Instruments Corp., Tarrytown, New York 10591]; Ektachem 700 Analyzer [tradename of Eastman Kodak Co., Rochester, New York 14650, USA); Astra [trademark of Beckman Instruments Inc., Brea, California 92621, USA]). Attention was soon drawn to the BUN analysis methods when it was discovered that one patient's serum had a BUN value of 950 mg/l on the Hitachi 737 and 370 mg/l on the 705! In addition, both instruments recorded creatinine values of 13 mg/l. The marked difference in BUN values and the unusual BUN-to-creatinine ratios on two instruments using similar chemical methods prompted this study. As a result of the study, such erroneous discrepancies have been eliminated by developing a new reagent system and a kinetic method of analysis for the two Hitachi analysers.

### Case report

A 73-year-old male who appeared healthy was admitted for a minor surgical procedure involving the removal of a small, metallic foreign body from his hand. A profile of common chemistry tests were performed and all were within normal limits with the exception of the BUN. Table 1 lists the patient's results from the Hitachi 737. Because of the unexplained and unusual BUN to creatinine ratio, the patient's operation was postponed while these results were compared with analyses by different chemical methods and the exact nature of the problem was discovered.

*Table 1. General chemistry results.*

| Test | Results | Units | Normal range |
|---|---|---|---|
| Gluc | 940 | mg/l | 700–1100 |
| BUN | 950 | mg/l | 80–230 |
| Creat | 13 | mg/l | 8–15 |
| Uric | 65 | mg/l | 36–64 |
| Na | 142 | nmol/l | 135–145 |
| K | 4·7 | mmol/l | 3·4–5·0 |
| CL | 108 | mmol/l | 95–108 |
| $CO_2$ | 27 mmol/l | | 24–35 |
| Ca | 92 | mg/l | 90–106 |
| Phos | 34 | mg/l | 25–45 |
| TP | 77 | g/l | 60–80 |
| Alb | 37 | g/l | 30–50 |
| Alkp | 60 | U/l | 30–130 |
| Ast | 14 | U/l | 10–45 |
| Alt | 10 | U/l | 10–45 |
| LD | 160 | U/l | 25–200 |
| Tbil | 7 | mg/l | 0–12 |
| Dbil | 2 | mg/l | 0–2 |
| Chol | 2010 | mg/l | 1500–2500 |
| Trig | 1210 | mg/l | 100–1700 |

### Materials and methods

*Boehringer Mannheim–Hitachi method*

Two separate reagents, R1 NS R2 (cat. Nos. 804550 and 804568 respectively for the Hitachi 737 and cat. No. 620400 for both reagents on the Hitachi 705; Boehringer Mannheim Diagnostics) were used. R1 contains NADH as the reactive ingredient together with an unspecified buffer and filler, which were defined by the manufacturer as nonreactive ingredients [2]. R2 contains urease, GLDH, and 2-oxogluarate in addition to an unspecified buffer. On both instruments, serum or calibrator is mixed with R1 and incubated for several minutes. At the end of this incubation period an initial bichromatic absorbance is measured as the difference between 376 nm and 660 nm or between 376 nm and 415 nm on the Hitachi 737 and 705, respectively. R2 is then added, mixed, and incubated for several additional minutes before a second bichromatic absorbance measurement is taken. The BUN concentration is proportional to the difference between the first and second absorbance measurements after a reagent blank is subtracted. The method is based on a two-stage reaction in which urea is hydrolysed by urease to form ammonia and $CO_2$.

$$\text{urea} + H_2O + 2\,H^+ \xrightarrow{\text{urease}} 2\,NH_4^+ + CO_2.$$

In the presence of GLDH and NADH, $NH_4^+$ reacts with 2-oxoglutarate to form L-glutamate and $NAD^+$

$$NH_4^+ + \text{2-oxoglutarate} + NADH \xrightarrow{\text{GLDH}} \text{L-glutamate} + NAD^+ + H_2O.$$

*Table 2. Reagent composition.*

| Component | Amount | Units |
|---|---|---|
| *Reagent 1* | | |
| Tris buffer | 60 | mmol/l |
| Adenosine-5′-diphosphate | 1·5 | mmol/l |
| NADH | 0·27 | mmol/l |
| Urease (Jack bean) | 25,000 | U/l |
| Glutamate dehydrogenase (Bovine liver) | 500 | U/l |
| pH=8 | | |
| *Reagent 2* | | |
| 2-oxoglutarate | 15 | mmol/l |

*Table 3. Chemistry parameters for Hitachi 737.*

| | | |
|---|---|---|
| Test name | : | BUN |
| Assay code | : | ENDP-11-20 |
| Sample volume | : | 4 µl |
| R1 Vol. | : | 300 µl |
| R2 | : | 100 µl |
| Wavelength 1 | : | 340 nm |
| Wavelength 2 | : | 415 nm |
| Compensate limit | : | 10·0 |
| Calibration | : | |
| Req. No. calib. | : | Conc. |
| 1) - - - Saline | : | 0 |
| 2) - - - Calib. | : | assigned value |
| Equation No. (1–5) | : | 1 |
| Factor (Fixed) | : | |
| Unit factor | : | 1·00 |
| Abs. limit (rate) | : | 0 |
| Inc/Dec | : | Dec |

- - - Denotes user or instrument specific settings.

### *Development of a kinetic BUN method*

Most commercial preparations for BUN reagents are available in single reagent vials. However, this convenience is generally offset by a shorter viable life as compared with two component reagent systems. Since both Hitachi instruments are capable of adding two reagents, a two-component reagent system was devised. The method is a modification of a single vial, kinetic BUN reagent produced by Reagent Applications. Reagent Applications prepared the first reagent, R1, with all components except 2-oxoglutarate, which they supplied in a second vial as R2 (cat. No. 85146, Reagent Applications, Inc., San Diego, California 92111, USA). The composition of each reagent is listed in table 2. The sample and all reagents, with the exception of 2-oxoglutarate, are preincubated. 2-oxoglutarate is added as a start reagent. Although the assay code for the Hitachi 737 specified an endpoint (ENDP), the manufacturer uses this nomenclature for two-point kinetic reactions. This code is used to take two separate absorbance readings after the addition of the second reagent, R2. Tables 3 and 4 show the new instrument settings for the Hitachi 737 and 705, respectively. Both R1 and R2 are stored at 4 °C and are stable for at least 10 days.

*Table 4. Instrument setings for Hitachi 705.*

| | | |
|---|---|---|
| Test code | : | BUN |
| Assay code | : | Rate-19-24 |
| Sample volume | : | 4 µl |
| R1 Vol. | : | 300 µl |
| Wavelength 1 | : | 415 nm |
| Wavelength 2 | : | 340 nm |
| Rgt. blk. abs. | : | 0 |
| Rgt. blk. conc. | : | 0 |
| Std. conc. | : | - - - |
| Factor | : | 0 |
| Std. abs. allowance | : | 10% |
| Normal range L | : | - - - |
| Normal Range H | : | - - - |
| Abs. limit (Rate) | : | 10000 |
| Control Id. No. | : | - - - |

- - - Denotes user or instrument specific settings.

### Results

### *Analyses by different chemical methods*

The BUN was measured on the DuPont aca, Technicon SMAC, Kodak Ektachem 700, and Beckman Astra. The results are summarized in table 5. The DuPont aca method is a two-step kinetic enzymatic assay employing urease and GLDH in which the sample is preincubated with all reagents except 2-oxoglutarate, which is added as a start reagent. On the SMAC, a diacetyl monoxime method employing a dialysis step is used. The Ektachem 700 uses an enzymatic method with urease in a dry chemistry slide. The Astra uses an enzymatic reaction with urease and measures the rate of change in conductance. The BUN values from the aca, SMAC, Ektachem, and Astra were similar and compatible with the patient's history, physical condition, and creatinine values.

Since both the Hitachi instruments and aca use similar enzymatic reactions, the main differences between the two systems are (1) the preincubation conditions; and (2) the end point versus kinetic method for measuring absorbance changes. Any type of precipitation that might occur during the preincubation period and then dissolve upon the addition of the second reagent would produce a falsely elevated BUN by the Hitachi method but not by the kinetic method or diacetyl monoxime method on the SMAC.

*Table 5. Analysis of patient's serum by different methods.*

| Instrument | Method | Result |
|---|---|---|
| Hitachi 737 | Enzymatic-end point* | 950 mg/l |
| Hitachi 705 | Enzymatic-end point* | 370 mg/l |
| DuPont aca | Enzymatic-kinetic | 230 mg/l |
| Technicon SMAC | Diacetyl monoxime | 210 mg/l |
| Kodak Ektachem 700 | Enzymatic-end point | 200 mg/l |
| Beckman astra | Enzymatic-kinetic | 170 mg/l |
| Hitachi 737 | Proposed enzymatic-kinetic++ | 230 mg/l |
| Hitachi 705 | Proposed enyzmatic-kinetic++ | 220 mg/l |

* Boehringer-Mannheim Reagents.
++ Reagent Applications, Inc. Reagents.

To test this possibility, the patient's sample was mixed in a test-tube with appropriate proportions of the BMD reagent 1. A large amount of fine precipitation was observed forming as the serum contacted the reagent. When BMD reagent 2 was added and mixed the precipitate completely disappeared. The exact cause of the precipitation in BMD reagent 1 is unknown, since the composition of the R1 reagent is not given in the manufacturer's literature. It is known, however, that patients with abnormally high levels of monoclonal immunoglobulins are prone to precipitation [3]. A serum protein electrophoresis confirmed our suspicion: a monoclonal peak was observed in the gamma globulin region. Immunochemical analysis revealed in IgM level of 21 g/l. In addition, the patient was found to have an abundant amount of lambda light chains in his urine. The patient's physician was notified and he is currently undergoing a full medical evaluation to determine the exact aetiology of his abnormal protein.

### *Linearity*

Human serum pools containing very low and extremely high urea concentrations were mixed in known proportions and assayed in triplicate to determine the linearity of our method. It proved to be linear to at least 1000 mg/l on both instruments.

### *Precision*

For the Hitachi 737, the test for within-run precision was performed by repeated analyses of sera from two different patients. Patient 1: $N = 20$, $x = 68$ mg/l, SD = 4, and CV = 5·8%. Patient 2: $N = 20$, $x = 448$ mg/l, SD = 12. and CV = 2·7%. Day-to-day precision was determined by repeated daily analysis of two human-based commercial control sera. Level 1: $N = 30$, $x = 151$ mg/l, SD = 4·8, and CV = 3·2%. Level 2: $N = 30$, $x = 510$ mg/l, SD = 10, and CV = 2·0%. For the Hitachi 705, the within-run precision test was again performed by repeated analyses of sera from two different patients. Patient 3: $N = 20$, $x =$ 140 mg/l, SD = 3·9, and CV = 2·8%. Patient 4: $N = 20$, $x = 608$ mg/l, SD = 7·2, and CV = 1·2%. Day-to-day precision was determined by repeated daily analyses of two human-based commercial control sera. Level 1: $N =$ 112, $x = 150$ mg/l, SD = 10, and CV = 3·7%. Level 2: $N = 107$, $x = 490$ mg/l, SF = 9, and CV = 1·9%.

Correlation with a kinetic enzymatic method—randomly selected serum samples were analysed in parallel on the Hitachi 737 and on the COBAS FARA (Roche Analytical Instruments Inc., Nutley, New Jersey 07110, USA) with enzymatic reagents from Reagent Applications, Inc. The correlation statistics are $N = 80$, $y = 0{\cdot}983\,x + 10$, $r = 0{\cdot}999$, and $S_{y.x} = 14$. At a later time, parallel analyses were performed on the Hitachi 737 and 705 with randomly selected serum samples using the proposed enzymatic rate method on both instruments. For $N = 31$, $y = 0{\cdot}964\,x - 58$, $r = 0{\cdot}999$, and $S_{y.x} = 39$ where units are in mg/l.

### Discussion

Two additional patients have been found who produce falsely elevated BUN values due to precipitation in R1 when analysed on the Hitachi 737 and 705 with BMD reagents. It was interesting to note that each patient had a different form of multiple myeloma; one patient had an elevated IgG and the other an elevated IgA. The marked discrepancies between BUN values on the Hitachi 737 and 705 were puzzling at first because both instruments use similar reagents. It was discovered, however, that this discrepancy was due to a difference in bichromatic absorbance measurements made on the Hitachi 737 (376 nm and 660 nm) and on the Hitachi 705 (376 nm and 415 nm). Because a fine precipitate produces significantly less absorbance at 660 nm than at 415 nm, the difference in absorbance between 376 and 660 nm was much greater than that observed between 376 and 415 nm.

In all three cases studied, normal BUN values were obtained on both instruments by using the proposed method. The method has been in use for over six months without any problems. An additional advantage to the new method is the improved day-to-day precision compared to the original method using BMD reagents.

### References

1. Kaplan, A., In: Meites, S. (Ed) *Standard Methods of Clinical Chemistry* (Academic Press, New York), **5** (1965), 245.
2. Boehringer Mannheim Diagnostics BUN Procedure. #6-519-0785 (1985).
3. Alexander, N. M., Gattra, R., and Nishimoto, M., *Clinica Chimica Acta*, **100** (1980), 301.

